# Concurrent Training Increases Serum Brain-Derived Neurotrophic Factor in Older Adults Regardless of the Exercise Frequency

**DOI:** 10.3389/fnagi.2022.791698

**Published:** 2022-03-07

**Authors:** Ermilo Canton-Martínez, Iván Rentería, Patricia C. García-Suárez, José Moncada-Jiménez, Juan Pablo Machado-Parra, Fabio Santos Lira, David K. Johnson, Alberto Jiménez-Maldonado

**Affiliations:** ^1^Facultad de Deportes, Universidad Autónoma de Baja California, Ensenada, Mexico; ^2^Department of Health, Sports and Exercise Sciences, University of Kansas, Lawrence, KS, United States; ^3^Human Movement Sciences Research Center (CIMOHU), University of Costa Rica, San Jose, Costa Rica; ^4^Exercise and Immunometabolism Research Group, Department of Physical Education, Paulista State University, UNESP, Presidente Prudente, Brazil; ^5^Department of Neurology, University of California, Davis, Davis, CA, United States

**Keywords:** concurrent training, heart rate, brain derived neurotrophic factor, body fat, elderly

## Abstract

**Background:**

Human brain function declines with aging. In this sense, exercise-based interventions has a promising effect on brain plasticity for older adults. Serum brain-derived neurotrophic factor (BDNF) is a positive biomarker for brain neuroplasticity in healthy older adults also modified by exercise training. Selected features of the exercise prescription for improving brain health are missing; therefore, the aim of this study was to determine the effects of concurrent exercise training frequency on serum BDNF levels in healthy older adults.

**Methods:**

Nineteen volunteers (age: 65 ± 4 year; body mass index: 28.0 ± 4.5 kg/m^2^) completed either a three times/week (3-t/w) (*n* = 8) or five times/week (5-t/w) (*n* = 11) concurrent exercise program. The exercise program lasted 11 weeks and all exercise sessions were performed for 50 min at moderate intensity. Serum BDNF, body composition, cardiovascular, and physical fitness variables were assessed before and after the exercise training program.

**Results:**

Regardless of the group, the serum BDNF increased following the intervention (*p* < 0.001), and there were no significant group (*p* = 0.827) or interaction (*p* = 0.063) effects. The maximal oxygen consumption (VO2max) increased regardless of the group (*p* = 0.007), with a non-significant group (*p* = 0.722) or interaction (*p* = 0.223) effects. Upper- and lower-body strength increased in both groups (*p* = 0.003); however, there was no effect of the training frequency (*p* = 0.53). For the skeletal muscle mass, there was a trend in the interaction effect (*p* = 0.053). Finally, the body fat percentage was unchanged.

**Conclusion:**

Eleven weeks of combined exercise training increased serum BDNF levels in healthy older adults, a response independent of the training frequency. The overall fitness level improved similarly in both exercise groups. These data reveal that a minimal dosage of concurrent exercise enhance functional capacity and a brain health biomarker in older adults.

## Introduction

In humans, aging is a biological condition characterized by a progressive and unstable decline of physiological capacities and core molecular functions (Carmona and Michan, 2016). Immunosenescence (Kohut and Senchina, 2004; Tylutka et al., 2021) and body composition redistribution are deleterious changes observed during aging (Kohrt et al., 1992; St-Onge and Gallagher, 2010). Indeed, in older adults, it is known that these processes elicit high body adiposity stores along with the concomitant reduction in skeletal muscle mass compared to younger adults (Kohrt et al., 1992; Janssen and Ross, 2005). The age-related reduction on skeletal muscle mass has a direct association with muscle function loss (Akima et al., 2001). To address this problem, several guidelines for aging adults recommend to perform strength training regularly (Garber et al., 2011; Fragala et al., 2019). Thus, research has been conducted to identify the optimal exercise dosage to maintain muscle mass and function in older adults (Galvão and Taaffe, 2005; DiFrancisco-Donoghue et al., 2007; Bickel et al., 2011). The evidence suggests that resistance training performed regularly at least once per week provides enough stimulus to maintain the neuromuscular performance in older adults; nevertheless, the same exercise dose was insufficient to maintain an adequate muscle mass (Bickel et al., 2011). Similar findings were reported before (Galvão and Taaffe, 2005; DiFrancisco-Donoghue et al., 2007). Age-related reductions in brain volume are believed to have a direct, negative impact on functional ability and preserved independence (Jack et al., 1998; Erickson et al., 2010). The brain-derived neurotrophic factor (BDNF) is a biomarker of healthy brain function (Erickson et al., 2010; Peng et al., 2011). The BDNF is a member of the neurotrophin family of growth factors (Nofuji et al., 2008), and in the brain, is mainly expressed in the hippocampus (Katoh-Semba et al., 1997) and plays an essential role in the viability and function of neuronal cells (Funakoshi et al., 1993; Mowla et al., 2001). Studies in humans provide evidence that exercise increases BDNF production and secretion from the brain into the periphery in the healthy elderly population (Erickson et al., 2011; Forti et al., 2015). Exercise training is widely recognized as an effective treatment to reduce the age-related cognitive decline, similar to its impact on skeletal muscle mass maintenance (Dishman et al., 2006; Erickson et al., 2010). However, the proper exercise dose-response aimed at improving brain function has not been elucidated as it has been shown in studies of peripheral organs. Therefore, the purpose of the study was to compare two exercise training routines (three or five times for week) on serum BDNF, body composition and selected functional and physiological responses in healthy older adults. The potential data to achieve in the current work might be considered as background to establish and design further studies aimed to identify a threshold of concurrent exercise to strength the brain health in aged population.

## Materials and Methods

### Participants

Nineteen healthy older adults [age: 64.8 ± 4.3 year; body mass index (BMI): 28.0 ± 4.5 kg/m^2^] enrolled in the “Active Aging” community health program from Facultad de Deportes of Universidad Autónoma de Baja California were recruited in the present study. The volunteers received information about the aim of the study and read and signed an institutionally approved informed consent. Participants completed a medical history questionnaire and reported being free of any musculoskeletal, cardiovascular, mental health problems, cognitive impairment, and metabolic diseases. Volunteers were excluded from the study if any drug or alcohol abuse was reported. Once included in the study, the participants completed an exercise program either three times/week (3-t/w, *n* = 8) or five times/week (5-t/w, *n* = 11; Table 1). The experimental group assignment was made according to the preferred participant’s time availability. Participants of both experimental groups performed aerobic and strength training (combined) exercises (see exercise training program for details). The study protocol followed the Helsinki Declaration principles, was approved by the Bioethics and Biosecurity Committee Facultad de Medicina y Psicología of Universidad Autónoma de Baja California and was registered under the code 889/2020-2.

**TABLE 1:** Demographic characteristics of the participants.

Variable	Three times/week (*n* = 8)	Five times/week (*n* = 11)	*P* =
Age (years)	64.8 ± 4.3	64.7 ± 4.6	0.99
BMI (kg/m^2^)	25.2 ± 2.5	30.1 ± 4.7	0.01
Education (years)	11.7 ± 1.5	12.0 ± 2.5	0.80
Gender (% female) (m/f)	87.5 (1/7)	81.8 (2/9)	

*The data are presented as the mean ± SD.*

### Experimental Procedures

**FIGURE 1:**
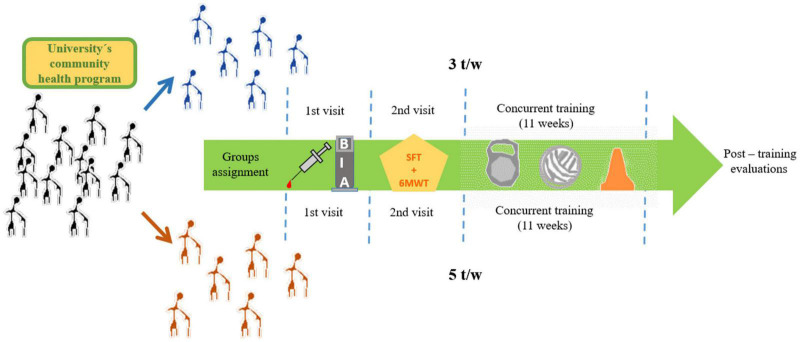
Experimental procedures.

This is a quasi-experimental study with baseline (pre-test) and post-training (post-test) measures of anthropometric, body composition, cardiovascular, functional capacity, and BDNF variables. The participants visited the Laboratorio de Fisiología Aplicada al Ejercicio Fisico twice for familiarization with the experimental procedures and the exercise training protocol. On the first visit, all participants underwent body composition evaluation and fasting venous blood sample collection for BDNF assessment. The second visit was scheduled 24-h before and after the concurrent exercise program for assessment of resting heart rate (HRrest), systolic blood pressure (SBP), diastolic blood pressure (DBP), mean arterial pressure (MAP), 6-min walking test (6MWT), and the Senior Fitness Test (SFT) (Figure 1).

### Anthropometric Assessment

The anthropometric assessments were performed on the first visit to the facilities. Body height (cm) was measured with an electronic stadiometer to the nearest ± 1 mm (Biospace Corporation, Seoul, South Korea). Body composition [i.e., body weight (kg), body fat mass (%), muscle mass (kg), and BMI] was measured with a bioimpedance analyzer Inbody™ model 770 (Inbody, Seoul, South Korea). The participants were instructed to refrain from eating or drinking for at least 2-h before anthropometric and body composition measurements were recorded.

### Cardiovascular and Physical Performance Assessment

During the second visit, the participants sat for 5-min, and then SBP and SBDP were recorded in mmHg. The SBP and DBP were measured in duplicate using an automatic sphygmomanometer Omron (OMRON Corp., Osaka, Japan). The MAP was estimated with the formula: DBP + (0.33 × [SBP-DBP]) (Sainas et al., 2016). Following the hemodynamic measurements, the 6MWT was performed following a standard procedure (Rikli and Jones, 1998; Guimarães et al., 2002). Briefly, participants wore a Tanita PD-733F pedometer (Tanita Corp., Arlington Heights, IL, United States) hanging from a strap to the neck and set to display the steps. Then, a member of the investigator team followed the manufacturer’s instructions to determine the participant’s stride length. All participants were instructed to walk around a basketball field for 6 min; the aim was to cover the longest possible distance without jogging or running. A member of the investigator team walked alongside the participants encouraging them to walk as fast as possible. Immediately after the 6MWT, the researcher registered the steps recorded to estimate the distance achieved (m). Throughout the 6MWT, all participants wore a chest strapped heart rate monitor Polar FT1 (Polar Electro, Kempele, Finland) for telemetric heart rate monitoring. Finally, the maximal oxygen consumption (VO2max) was estimated following the equation reported by Burr (Burr et al., 2011). After the 6MWT, the participants completed the Senior Fitness Test (Jones and Rikli, 2002).

### Blood Collection and Brain-Derived Neurotrophic Factor Analysis

A resting 4-mL venous blood was drawn from the antecubital vein of the non-dominant arm of the participant by a certified phlebotomist. Blood samples were drawn into 6 mL BD Vacutainer 368175 tubes (Becton, Dickinson and Co., Franklin Lakes, NJ, United States) with no additives for serum collection. Blood was allowed to clot at room temperature for 40-min. Then, the tubes were centrifuged at 1,500 rpm for 10-min at room temperature and the supernatant was isolated and stored into 1.5 mL Eppendorf tubes at −20°C until further analysis. Serum samples were diluted 1:10 with dilution buffer from the manufacture’s kit and BDNF’s concentration was obtained by an enzyme-linked immunoassay (ELISA) kit Abcam ab99978-BDNF (Abcam, Cambridge, United Kingdom) following the manufacturer’s instruction. Detection limits were <80 pg/mL, mean inter-and intra-assay coefficients of variance were <10 and <12%, respectively. The absorbance was read at 450 nm in an iMark microplate absorbance reader BIO-RAD Cat. 168-1130 (Bio-Rad Laboratories, Inc., Hercules, CA, United States), immediately after the stop solution was added.

### Exercise Training

The exercise training protocol performed by the participants consisted of the same duration and intensity; the only difference was the weekly frequency (i.e., 3-t/w vs. 5-t/w). The training program was comprised of aerobic and resistance training exercises; the aerobic component included walking on a flat surface, walking up and down a flight of stairs, and skipping exercise performed for 50-min. The resistance exercise component included chair stands for the lower body, and biceps and triceps curls, horizontal adduction, and overhead press for the upper body. All the upper-body exercises were performed with elastic bands for 50-min. The exercise intensity for each exercise session was monitored with the 6–20-point Borg’s rating of perceived exertion (RPE) every 10-min throughout the session. Exercises were adjusted to reach a RPE between 12 and 14 points (Supplementary Figure 1). Furthermore, each exercise session included a warm-up and cool-down recovery including passive stretch exercises for 10-min. The length of the training program was 11 weeks for both experimental groups; all sessions were performed between 08:00 and 10:00 a.m. and were supervised by the investigator team members.

### Post-training Procedures

Following the completion of the 11-week exercise program, all anthropometric, body composition, blood samples, cardiovascular, SFT, and 6MWT measurements were performed using the same procedures described above for baseline (Figure 1).

### Statistical Analysis

The statistical analyses and figures were completed with the GraphPad Prism 6.0 software (Graphpad Holdings, LLC., La Jolla, CA, United States) and JASP statistical software v. 0.14.1 (University of Amsterdam, Amsterdam, Netherlands). Descriptive statistics are presented as the mean and standard deviation (M ± SD). Independent-samples *t*-tests compared participant’s demographic, age, BMI, and education years at baseline. Serum BDNF concentration, cardiovascular, SFT, and anthropometric variables were analyzed with a two-way repeated-measures ANOVA (group × time) followed by Bonferroni’s *post hoc* tests when appropriate. We used the Kolmogorov-Smirnov normality test to analyze the dependent variables. Based on the results, we identified only three variables that departed from the normality assumption; however, ANOVA is considered a robust test that tolerates violations to its normality assumption (Blanca et al., 2017). The η^2^ effect size was computed and interpreted as small (≤0.01), medium (0.06), and large (≥0.14) (Cohen, 1992). Statistical significance was set *a priori* at *p* ≤ 0.05.

## Results

### Characteristics of the Participants

Participant characteristics at baseline by experimental group are summarized in Table 1.

### Effect of Exercise Frequency on Serum Brain-Derived Neurotrophic Factor Levels

**FIGURE 2:**
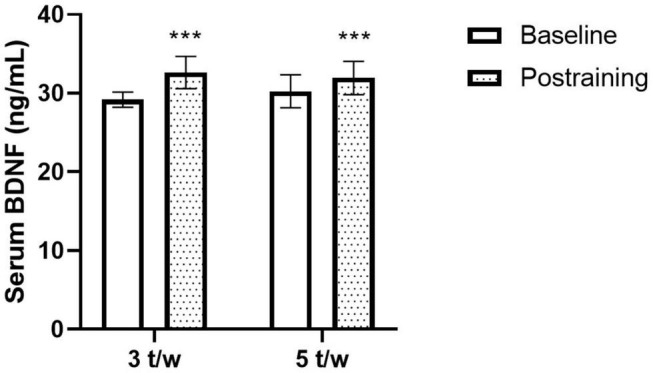
Effects of the exercise training frequency on serum brain-derived neurotrophic factor (BDNF) levels. The chronic concurrent exercise program increased the peripheral BDNF levels in healthy older adults. The response was independent of the exercise training frequency. Two-way ANOVA. ***p < 0.05 vs. Baseline. Data are Mean ± SD.

The effects of the two exercise training frequencies on serum BDNF are shown in Figure 2. Three participants of the 5-t/w group were excluded from the analysis due to negative absorbance values. There was a small trend for the interaction effect (*p* = 0.063; η^2^ = 0.039), non-significant group main effect (*p* = 0.827; η^2^ = 0.002), and a significant time main effect (*p* < 0.001; η^2^ = 0.328) on the BDNF concentration.

### Effects of Exercise Frequency on Cardiovascular, Fitness and Body Composition Markers

**FIGURE 3:**
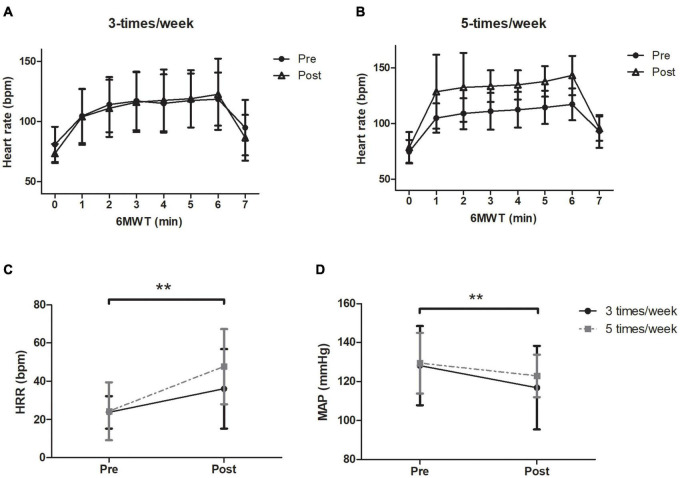
Heart rate response through the 6-min walking test (6MWT) and during 1 min immediately finished the exercise testing. **(A)** 3-t/w and **(B)** 5-t/w groups. Absolute values of heart rate recovery during the first minute of immediately finished the 6MWT **(C)**. Baseline mean arterial pressure (MAP) values before and after exercise program **(D)**. Two-way ANOVA. **p < 0.05 vs. Baseline. Data are Mean ± SD.

The heart rate response during the 6MWT before and after the exercise program for the 3- and 5-t/w groups are presented in Figures 3A,B, respectively. There was an interaction between group and time on HRrest (*p* = 0.042; Table 2). The follow-up analysis showed a reduction trend (*p* = 0.075) in the HRrest in the 3-t/w exercise group compared to the pre-test scores. For peak heart rate (HRpeak) during the 6MWT there was a time main effect (*p* = 0.024; η^2^ = 0.111), and a medium effect for interaction (*p* = 0.090; η^2^ = 0.06; Table 2). A main time effect was observed for the heart rate recovery (HRR) (*p* = 0.003 η^2^ = 0.222; Figure 3C). There were main time effects for MAP (*p* = 0.002; η^2^ = 0.07; Figure 3D), DBP (*p* = 0.003; η^2^ = 0.074), and VO2max (*p* = 0.007; η^2^ = 0.48; Table 2).

**TABLE 2:** Hemodynamic variables at baseline and post-training.

Variable	Three times/week (*n* = 8)		Five times/week (*n* = 11)		*P*≤			
	Baseline	Post-training	Baseline	Post-training	Time effect	Group effect	Interaction effect	η^2^ for interaction
HRrest (bpm)	80.88 ± 14.57	73.38 ± 7.89	74.64 ± 10.62	78.55 ± 13.78	0.499	0.916	0.042	0.221
HRpeak (bpm)	118.50 ± 22.0	117.30 ± 14.30	122.60 ± 29.60	143.20 ± 17.7	0.023	0.210	0.089	0.059
HRR 1 min (bpm)	23.75 ± 8.51	36.00 ± 20.79	24.27 ± 15.14	47.72 ± 19.66	0.003	0.322	0.282	0.022
SBP (mmHg)	138.88 ± 26.94	131.88 ± 21.53	137.64 ± 18.78	134.64 ± 15.76	0.218	0.931	0.615	0.015
DBP (mmHg)	81.88 ± 16.08	72.88 ± 18.13	83.55 ± 11.00	78.00 ± 8.16	0.003	0.566	0.421	0.038
MAP (mmHg)	128.16 ± 20.32	116.83 ± 21.42	129.42 ± 15.57	122.87 ± 10.90	0.002	0.631	0.342	0.005
VO2max (ml⋅kg^–1^/min^–1^)	28.96 ± 3.030	31.75 ± 2.84	29.04 ± 3.96	30.21 ± 6.12	0.007	0.722	0.224	0.086
RPP (units/10^–3^)	11.13 ± 2.58	9.75 ± 2.32	10.28 ± 1.98	10.61 ± 2.45	0.266	0.996	0.076	0.174

*Data are M ± SD. SBP, systolic blood pressure; DBP, diastolic blood pressure; MAP, mean arterial pressure; HRR, heart rate recovery; RPP, rate pressure product.*

For the body composition variables, a group effect was observed in fat mass (%) (*p* = 0.037; η^2^ = 0.230). A significant time (*p* = 0.007; η^2^ = 0.03) and a small interaction effect between group and time (*p* = 0.053; η^2^ = 0.013) was found in skeletal muscle mass (Table 3). Regarding the fitness levels, a significant main interaction effect was found on the 6MWT distance (*p* = 0.031; η^2^ = 0.04). Only the participants in the 5-t/w group walked a longer distance during the 6MWT following the exercise program (*p* < 0.001; η^2^ = 0.233; Table 3). Regardless of the group, a time effect was found on the lower-body strength (*p* = 0.003; η^2^ = 0.146), the upper-body strength (*p* = 0.003; η^2^ = 0.118), and the agility (*p* = 0.001). No significant group (*p* = 0.537) and interaction (*p* = 0.135) effects were observed on agility (Table 3).

**TABLE 3:** Body composition and physical performance (SFT and 6MWT) of the experimental groups.

Variable	Three times/week (*n* = 8)		Five times/week (*n* = 11)		*P*≤			
	Baseline	Post-training	Baseline	Post-training	Time effect	Group effect	Interaction effect	η^2^ for interaction
Body weight (kg)	67.94 ± 5.80	66.83 ± 7.25	71.70 ± 12.92	73.81 ± 11.87	0.612	0.070	0.113	0.141
BMI (kg/m^2^)	25.23 ± 2.51	25.29 ± 2.71	30.09 ± 4.72	30.42 ± 4.93	0.273	0.017	0.452	0.034
Skeletal muscle mass (kg)	22.40 ± 3.11	22.80 ± 3.49	22.73 ± 3.80	24.76 ± 3.60	0.007	0.485	0.053	0.203
Fat mass (%)	23.31 ± 5.93	23.11 ± 6.02	31.47 ± 8.84	31.58 ± 9.28	0.912	0.037	0.708	0.008
Chair stand (rep)	14.63 ± 3.66	16.88 ± 3.91	13.82 ± 3.28	16.82 ± 2.43	0.003	0.749	0.630	0.014
Arm curls (rep)	14.38 ± 3.50	16.25 ± 3.88	14.82 ± 2.93	17.82 ± 3.52	0.003	0.494	0.428	0.037
Flexibility (cm)	1.13 ± 6.73	2.38 ± 3.85	1.00 ± 4.86	3.27 ± 3.00	0.168	0.833	0.681	0.010
Agility (s)	6.24 ± 1.15	5.31 ± 0.50	6.44 ± 1.42	4.65 ± 0.49	0.001	0.537	0.135	0.126
6MWT distance (m)	498.47 ± 60.12	543.71 ± 53.20	477.88 ± 84.77	586.65 ± 73.04	0.001	0.714	0.031	0.247

*Data are M ± SD.*

## Discussion

Eleven weeks of concurrent (i.e., aerobic + strength) exercise induced positive changes for fitness and strength and increased serum BDNF; however, the increment of circulating BDNF was independent of the exercise training frequency; similar responses were observed for variables associated with functional capacity. The BDNF is a neurotrophin sensitive to changes elicited by chronic aerobic, strength, or concurrent exercise (Seifert et al., 2010; Schmolesky et al., 2013; Dinoff et al., 2016). The positive impact of acute concurrent exercise session on circulating BDNF has been reported in the young population (Murawska-Cialowicz et al., 2015; Figueiredo et al., 2019). However, there are contradictory findings in older adults (Kim et al., 2014; Nascimento et al., 2014; Ruiz et al., 2015). For instance, there is evidence reporting non-significant effects of exercise on BDNF (Ruiz et al., 2015), whereas others have found high circulating BDNF levels following a long-term concurrent training (Nascimento et al., 2014). Our results agree with the latter (Figure 2). It is worthy to notice that the age of the participants in the study by Nascimento et al. (66 year) and the current work (64 year) was similar; however, the participants in the study by Ruiz et al. were older (92 year). Therefore, it seems that age could be a moderator variable associated with the BDNF regulatory response to a concurrent exercise program. In line with this, other authors agreed that aging is a biological regulator of peripheral BDNF concentration in humans (Lommatzsch et al., 2005; Driscoll et al., 2012). The exercise program prescribed in the current study did not require exercise equipment (i.e., bench press machine, Smith machine); a similar exercise model was reported by others (Nascimento et al., 2014). The previous information emphasizes the efficacy of an exercise program free of expensive equipment to increase the serum BDNF in healthy older adults. Considering this, others have previously demonstrated the efficacy of exercise programs free of expensive equipment to improve cognitive performance in healthy older adults (Eckardt et al., 2020).

There is a plethora of potential molecular mechanisms elicited by combined exercise aimed at increasing the serum BDNF. First, the exercise program prescribed in the present study included moderate-intensity aerobic exercise. It has been suggested -and partially demonstrated-, that aerobic exercise increases the neuronal activity that increases intracellular calcium, which further enhances BDNF synthesis and release (Fernandes et al., 2017). The exercise program also included strength exercises, and recent meta-analytical evidence shows that resistance training exercise increases insulin-like growth factor-1 (IGF-1) synthesis in skeletal muscle in male and female older adults (Jiang et al., 2020; Ye et al., 2021). This anabolic molecule reaches the brain to induce BDNF production (Herold et al., 2019; Pinho et al., 2019).

Additionally, the lactate generated during exercise is another potential molecule to enhance the synthesis of BDNF in brain; consequently, increasing the BDNF concentration in the periphery (Hashimoto et al., 2021; Huang et al., 2021). In this sense, resistance training in older people is a stimulus that increase blood lactate in healthy elderly (Valkeinen et al., 2006; Simões et al., 2013). Therefore, we did not discard that the training program applied in the current work increased the blood lactate, facilitating the raising of serum BDNF. Finally, the BDNF measured in serum is released during by the platelets during their activation (Gejl et al., 2019); that response happened because the platelets (blood cells) are the main reservoir of the circulating neurotrophin (Fujimura et al., 2002). Recent evidence showed that chronic physical exercise did not modify the function of the platelets in healthy elderly population (Haynes et al., 2018). Therefore, we discard that the changes in the serum BDNF showed by the participants can be derived from platelet function more than the brain adaptation to exercise.

The results of the present study showed that there was no significant effect of the training frequency. To the best of our knowledge, this is the first study aimed to determine the impact of different concurrent exercise frequencies on serum BDNF in healthy older adults. Others have studied the effects of different exercise intensities on serum BDNF in a similar population (Forti et al., 2015). For instance, Forti et al. (2015) found that the exercise intensity significantly changed circulating BDNF in males but not in females. In the present study, there was a high proportion of women compared to men (Table 1); therefore, we do not discard the possibility of the majority of female participants in our sample could become a potential factor attenuating the changes in BDNF. Further studies are needed to clarify this effect.

With regard to the cardiovascular adaptations, the HRR, HRpeak, and MAP improved after the concurrent exercise program (Table 2). The attenuated HR response to exercise, named chronotropic incompetence, has been identified as an independent risk marker of mortality (Lauer et al., 1999; Khan et al., 2005). Indeed, the chronotropic incompetence becomes more pronounced with aging, which is prevented by appropriate cardiorespiratory fitness (Ciolac et al., 2014; Ozemek et al., 2016). The high HRpeak found in the current work indicates the efficacy of the concurrent exercise program to improve heart function during exertion (i.e., 6MWT). The greater HRR after the intervention completion reinforces the positive impact of concurrent exercise to strengthen the cardiovascular health, mainly the cardiac function in older adults. Together with the changes mentioned above, the VO2max was also improved with the combined exercise program (Table 2). Our findings agree with other studies with similar duration (Campos et al., 2013; Chapman et al., 2013). The increased distance achieved on the 6MWT by the 5-t/w group after the intervention suggests that the improvement of the VO2max can be dependent on the training frequency in older adults. We consider the null magnitude of VO2max observed between groups in the current study is explained by the indirect estimation method employed (Burr’s equation). Further studies are needed to rectify the lowest amount of combined exercise (i.e., concurrent) frequency to increase VO2max in older adults.

The upper- and lower-limb strength improved after 11 weeks of concurrent exercise. Our findings agree with others using a similar exercise prescription (i.e., 12-week duration, 3-t/w) (Campos et al., 2013). In addition to the increment of segmental strength, agility was also improved (Table 3). However, there was not a significant difference in functional capacity variables between groups after the intervention (Table 3). Our findings are consistent with others who reported no significant effects of the training frequency on the functional capacity in healthy older adults (Taaffe et al., 1999; Turpela et al., 2017). The previous information highlights the potential application of concurrent exercise programs for sedentary older adults. The proposal of strength and aerobic training carried out three times per week for ∼50-min helps to maintain the brain and cardiovascular health during aging.

Finally, for the skeletal muscle mass, there was a strong trend of the interaction effect, having the 5-t/w the group with more skeletal muscle mass increase compared to the 3-t/w group after 11 weeks of training. Body weight, BMI, and body fat percentage were unaffected by the exercise program (Table 3). The results regarding body composition are unclear; some components showed a trend for improvement whereas the variables associated with obesity did not change. The current findings agree with previous studies that used chronic exercise interventions (Taaffe et al., 1999; Murlasits et al., 2012; Campos et al., 2013; Turpela et al., 2017; Marcos-Pardo et al., 2019). In these studies, including the current work, the diet was not controlled or accounted for. The previous information leads us to consider the diet as a key variable to modify the body composition in healthy older adults. Our study has important limitations. One of them is the small sample size analyzed, even though we recruited small participants in our work, similar studies did involve similar sample sizes (Kim et al., 2014; Ruiz et al., 2015). Another limitation was that the work did not included a non-exercise control group. The study was carried out in a community health program focused to promote the regular exercise practice in elderly. Therefore, the limit or reduce the participation of elderly to recruit a control group will be have ethical issue for the community outreach program. A second limitation is that our work did not included cognitive testing. Although the BDNF is considered a direct marker of brain health (mainly hippocampus function in elderly) (Erickson et al., 2010), this neurotrophin only cover one of the multiple levels to analyze the neurocognitive function (Stillman et al., 2016; Herold et al., 2019), studies employing techniques to determine the structural brain changes and cognitive testing are needed to clarify the impact of the concurrent training on neurocognitive function in healthy elderly population.

## Conclusion

The current work demonstrates that a moderate-length concurrent exercise training program increases fasting serum BDNF levels in healthy older adults. This response seems independent of the training frequency, which suggests a plateau for the CNS response to the concurrent exercise stimulus. The present evidence emphasizes the unnecessary excessive training to achieve a healthier brain in older adults. Moreover, the physical fitness level was improved in the same magnitude in both exercise groups. These data reveal a minimal dose-response of concurrent exercise to improve functional capacity in older adults.

## Data Availability Statement

The raw data supporting the conclusions of this article will be made available by the authors, without undue reservation.

## Ethics Statement

The studies involving human participants were reviewed and approved by Bioethics and Biosecurity Committee Facultad de Medicina y Psicología of Universidad Autónoma de Baja California and was registered under the code 889/2020-2. The patients/participants provided their written informed consent to participate in this study.

## Author Contributions

AJ-M, IR, and EC-M: conceptualization. PG-S, JM-P, and EC-M: methodology. JM-J and PG-S: statistical analysis. AJ-M: writing—original draft preparation. FL and DJ: writing—review and editing. PG-S, IR, and JM-J: manuscript preparation and editing. All authors have read and agreed to the published version of the manuscript.

## Conflict of Interest

The authors declare that the research was conducted in the absence of any commercial or financial relationships that could be construed as a potential conflict of interest.

## Publisher’s Note

All claims expressed in this article are solely those of the authors and do not necessarily represent those of their affiliated organizations, or those of the publisher, the editors and the reviewers. Any product that may be evaluated in this article, or claim that may be made by its manufacturer, is not guaranteed or endorsed by the publisher.

## Supplementary Material

Supplementary Figure 1 Values of the rating of perceived exertion (Borg scale-20) for each exercise training session. Data are M ± SD.
