# Supplementary figures and images for: Concurrent Training Increases Serum Brain-Derived Neurotrophic Factor in Older Adults Regardless of the Exercise Frequency
Source: Front Aging Neurosci. 2022 Mar 7;14:791698. doi: 10.3389/fnagi.2022.791698 (PMC8940272; doi:10.3389/fnagi.2022.791698)

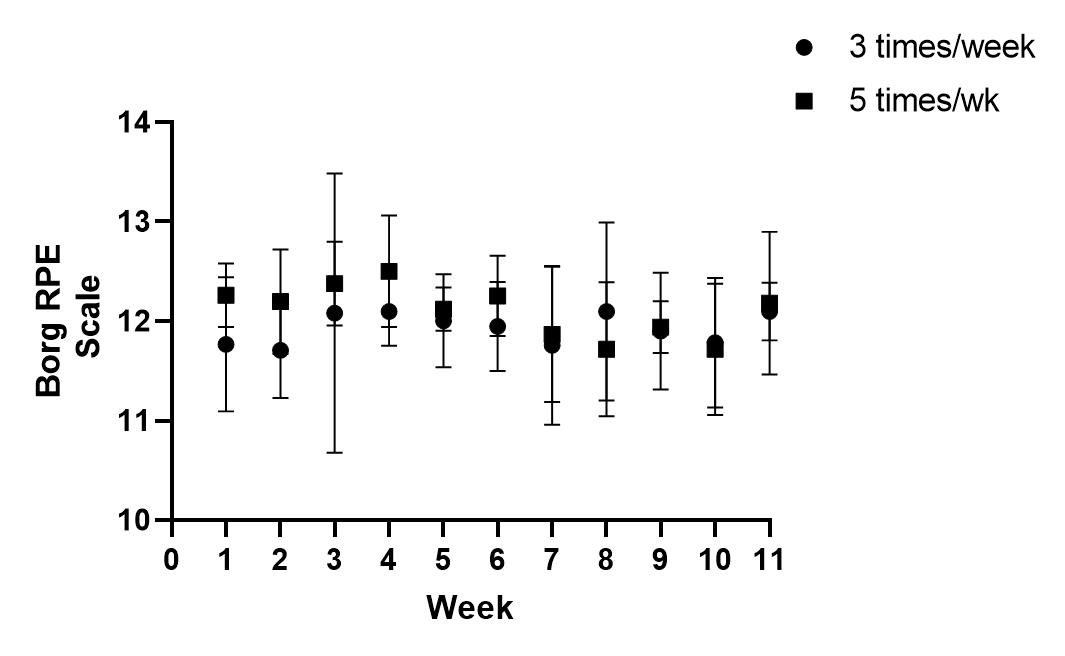

Supplement: Supplementary Figure 1 — Values of the rating of perceived exertion (Borg scale-20) for each exercise training session. Data are M ± SD. [file Image_1.TIF]
